# Optical Constants and Structural Properties of Epitaxial MoS_2_ Monolayers

**DOI:** 10.3390/nano11061411

**Published:** 2021-05-27

**Authors:** Georgy A. Ermolaev, Marwa A. El-Sayed, Dmitry I. Yakubovsky, Kirill V. Voronin, Roman I. Romanov, Mikhail K. Tatmyshevskiy, Natalia V. Doroshina, Anton B. Nemtsov, Artem A. Voronov, Sergey M. Novikov, Andrey M. Markeev, Gleb I. Tselikov, Andrey A. Vyshnevyy, Aleksey V. Arsenin, Valentyn S. Volkov

**Affiliations:** 1 Center for Photonics and 2D Materials, Moscow Institute of Physics and Technology, 9 Institutsky Lane, 141700 Dolgoprudny, Russia; georgiy.ermolayev@phystech.edu (G.A.E.); mira@phystech.edu (M.A.E.-S.); dmitrii.yakubovskii@phystech.edu (D.I.Y.); voronin.kv@phystech.edu (K.V.V.); mihailtatmyshev@inbox.ru (M.K.T.); doroshina.nv@phystech.edu (N.V.D.); nemtsov@phystech.edu (A.B.N.); voronov.aa@mipt.ru (A.A.V.); novikov.s@mipt.ru (S.M.N.); markeev.am@mipt.ru (A.M.M.); tselikov.gi@mipt.ru (G.I.T.); andrey.vyshnevyy@phystech.edu (A.A.V.); arsenin.av@mipt.ru (A.V.A.); 2 Department of Physics, Faculty of Science, Menoufia University, Shebin El-Koom 32511, Egypt; 3 Skolkovo Institute of Science and Technology, 3 Nobel Street, 143026 Moscow, Russia; 4 Moscow Engineering Physics Institute, National Research Nuclear University MEPhI, 31 Kashirskoe Sh., 115409 Moscow, Russia; limpo2003@mail.ru; 5 GrapheneTek, Skolkovo Innovation Center, 143026 Moscow, Russia

**Keywords:** transition-metal dichalcogenides, MoS_2_ monolayer, molecular beam epitaxy, optical constants, dielectric properties, refractive index, nanophotonics, spectroscopic ellipsometry

## Abstract

Two-dimensional layers of transition-metal dichalcogenides (TMDs) have been widely studied owing to their exciting potential for applications in advanced electronic and optoelectronic devices. Typically, monolayers of TMDs are produced either by mechanical exfoliation or chemical vapor deposition (CVD). While the former produces high-quality flakes with a size limited to a few micrometers, the latter gives large-area layers but with a nonuniform surface resulting from multiple defects and randomly oriented domains. The use of epitaxy growth can produce continuous, crystalline and uniform films with fewer defects. Here, we present a comprehensive study of the optical and structural properties of a single layer of MoS_2_ synthesized by molecular beam epitaxy (MBE) on a sapphire substrate. For optical characterization, we performed spectroscopic ellipsometry over a broad spectral range (from 250 to 1700 nm) under variable incident angles. The structural quality was assessed by optical microscopy, atomic force microscopy, scanning electron microscopy, and Raman spectroscopy through which we were able to confirm that our sample contains a single-atomic layer of MoS_2_ with a low number of defects. Raman and photoluminescence spectroscopies revealed that MBE-synthesized MoS_2_ layers exhibit a two-times higher quantum yield of photoluminescence along with lower photobleaching compared to CVD-grown MoS_2_, thus making it an attractive candidate for photonic applications.

## 1. Introduction

The discovery of graphene as the first known 2D material [1] has generated a great momentum for research in nanoelectronics and nanophotonics based on low-dimensional materials [2,3,4,5]. Great efforts have been devoted to expanding the range of available materials. As a result, a new “periodic table” of 2D materials has been created, which comprises groups of transition metal chalcogenides (MX_n_) [6,7], hexagonal boron nitride [8], monatomic materials, such as silicene, germanene, phosphorene, borophene [9,10,11,12], and a family of MXenes [13,14].

The family of transition metal dichalcogenides (TMDs) was widely recognized for the diversity of their electronic properties, encompassing superconductors [15], conductors [16,17], and semiconductors [18,19]. In addition to this vast diversity, materials can be stacked on each other, thus forming van der Waals heterostructures [19] and attaining novel properties. Furthermore, even monolayers of the same material can change their properties drastically when stacked to form twisted bilayers [20]. Thus, the use of TMDs allows one to design a large number of electronic, nanophotonic, mechanical, and thermal devices based only on 2D materials [6,21,22,23,24], making them inherently flexible and easy to use.

MoS_2_ is a prominent representative of TMD semiconductors. When thinned down to a single atomic layer, it undergoes a transition from an indirect to a direct bandgap semiconductor [25], which is crucial for photonic applications as the radiative quantum yield drastically increases upon such transition [26]. In practice, monolayers of MoS_2_ are typically obtained either via exfoliation or chemical vapor deposition (CVD). Both methods have serious drawbacks. Although exfoliated flakes have excellent structural and optical properties, their size is limited by a few microns, which hinders their commercial applications. CVD MoS_2_ films overcome the size limitation, but at a price of decreased quality, both structural and optical. Recently, epitaxially grown films of TMDs [27], including MoS_2_ [28], with a thickness down to a single atomic layer, have become available. Molecular beam epitaxy is a mature technology for the production of atomically smooth monocrystalline thin semiconductor films [29]; therefore, it has the potential to overcome size and quality limitations of exfoliation and CVD, respectively. At the same time, while the properties of CVD MoS_2_ monolayers have been studied previously [30,31,32,33,34], little is known about the optical properties of available MBE-grown MoS_2_ monolayers. Although recent works [27,35,36] report optical and electronic properties of MBE TMDs close to the exfoliated one, their epitaxial samples has at a maximum 200 μm lateral size, which is insufficient for the majority of applications [2,3,4,5]. To resolve this limitation, we focused on a large-scale MBE MoS_2_, which covers more than 97% of the substrate surface.

Here we present a comprehensive study of the optical and structural properties of a MoS_2_ monolayer grown by MBE on a sapphire substrate. Optical properties were measured by spectroscopic ellipsometry (SE) in a broad spectral range from 250 to 1700 nm. Using the Accurion EP4 imaging ellipsometer, which is capable of collecting the signal from a small micrometer-scaled area, we have measured optical constants of epitaxially grown monolayer MoS_2_, and compared them with the available data of CVD-grown and exfoliated monolayer MoS_2_ [37,38]. The structural properties of MoS_2_ samples were assessed in a combined study comprising optical microscopy, scanning electron microscopy (SEM), atomic force microscopy (AFM), X-ray photoemission spectroscopy (XPS), Raman spectroscopy, and photoluminescence imaging. We find that MBE produces a polycrystalline monolayer film with a high crystallinity whose quantum yield of luminescence is higher than that of the CVD monolayers of MoS_2_.

## 2. Results and Discussion

### 2.1. Sample Preparation and Characterization

**Figure 1:**
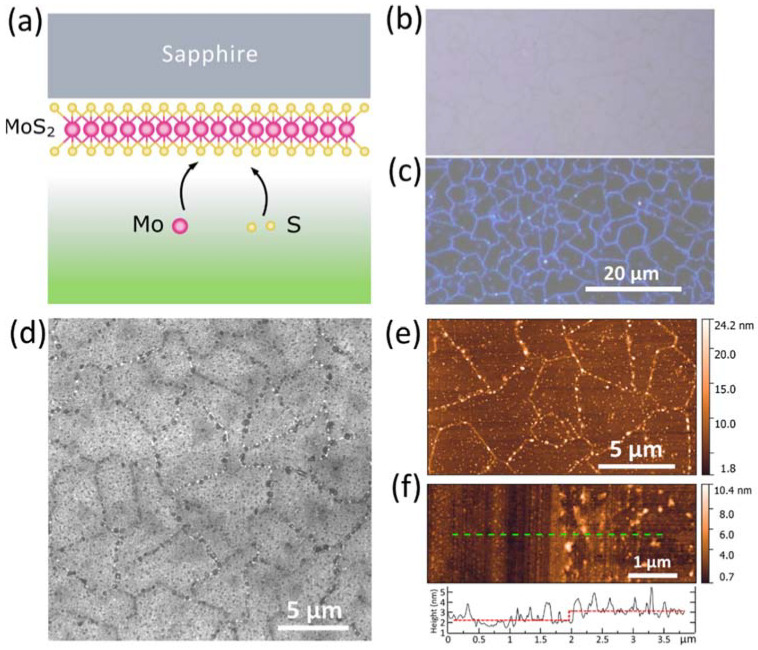
(**a**) A schematic diagram showing the concept of MBE growth monolayer MoS_2_ on a sapphire substrate. (**b**,**c**) Optical image of the epitaxial MoS_2_ taken in bright field (**b**) and dark field (**c**) regimes. MoS_2_ covers more than 97% of the surface. (**d**) SEM image of the epitaxial MoS_2_ revealing the high crystallinity of the samples with the characteristic crystallite size 6 ± 2 μm. (**e**) The AFM topography map of MoS_2_ surface with a scan area of 17.5 × 10 μm^2^. Root mean square roughness of MoS_2_ is 0.5 nm in areas without defects. (**f**) The AFM topography map and the cross-sectional profile of the edge of epitaxial MoS_2_ along the green line, giving the MoS_2_ layer thickness of ~0.9 nm. The scan area was 5.5 × 2 μm^2^.

Monolayers of MoS_2_ were prepared through molecular beam epitaxy as schematically shown in Figure 1a. Before measurement, MoS_2_ samples were washed and annealed in a vacuum chamber to remove any contaminants. The measured samples were uniform and high-crystalline as confirmed by images from optical, scanning electron microscopy (SEM), and atomic force microscopy (AFM) shown in Figure 1b–e. The epitaxial MoS_2_ monolayer uniformly covers the double polished sapphire substrate with an average crystallite size of 6  μm, confirming the high quality of the samples [39]. Next, we validated that grown MoS_2_ is atomically thin using AFM. The measured topography in Figure 1f yields 0.9  nm for film thickness, which is consistent with the previous results for monolayer MoS_2_ [23,40,41,42,43].

**Figure 2:**
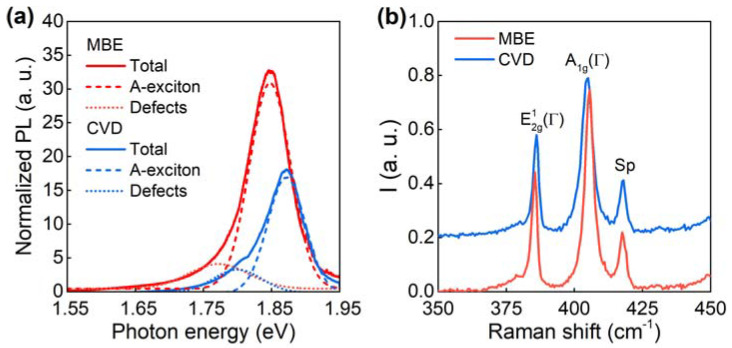
Photoluminescence (**a**) and Raman (**b**) spectra of the CVD and epitaxial monolayer MoS_2_ grown on sapphire substrates. The excitation wavelength was 632.8 nm (**a**) and 532 nm (**b**). Dashed and dotted lines show deconvolution of the photoluminescence spectrum into Gaussian peaks corresponding to A-exciton and defects, respectively. The Raman peak marked as “Sp” is related to the sapphire substrate.

We carried out a detailed comparison of photoluminescence and Raman spectra for epitaxial and CVD-grown MoS_2_ monolayers for further investigation of the samples’ quality. Photoluminescence (PL) of the CVD and epitaxial monolayer MoS_2_ grown on sapphire substrates are presented in Figure 2a. PL was excited resonantly at 632.8 nm. Each spectrum was deconvoluted into two Gaussian peaks with maxima at about 1.87 and 1.8 eV in the case of CVD-grown MoS_2_ and 1.85 and 1.78 eV in MBE-synthetized MoS_2_. The first peak A corresponds to the radiative excitonic recombination while the microscopic origin of the second peak remains a disputable topic. Earlier works attribute it to negatively charged trions [44,45] related to n-type conductivity, while a more recent work [46] argues that it stems from the recombination of bound excitons formed on either the unintended impurities or the native point defects. Regardless of the PL mechanism (bound excitons or trions) of the peak, in both cases it comes from structural defects of the sample, since inherent n-type conductivity can originate only from defects [47] or donor impurities. Hence, the defect’s contribution into PL spectrum is 14% for epitaxial MoS_2_ and 22% for CVD MoS_2_, thereby validating the lower density of structural defects in MBE MoS_2_. Moreover, A-exciton PL is almost two times brighter for MBE MoS_2_ compared to the CVD sample, as is clearly seen in Figure 2a. Additionally, PL from epitaxial MoS_2_ has a 0.025 eV red-shift in respect to the CVD sample, which implies that MBE MoS_2_ has a slightly different crystal structure. The non-resonant Raman scattering spectra of the CVD and the epitaxial monolayer MoS_2_ grown on sapphire substrates are presented in Figure 2b. The value of frequency difference between the $A_{1g}(\Gamma )$ and $E_{2g}^{1}(\Gamma )$ modes equals 20 cm^−1^ for both samples. This value confirms the monolayer nature of both studied samples [48].

**Figure 3:**
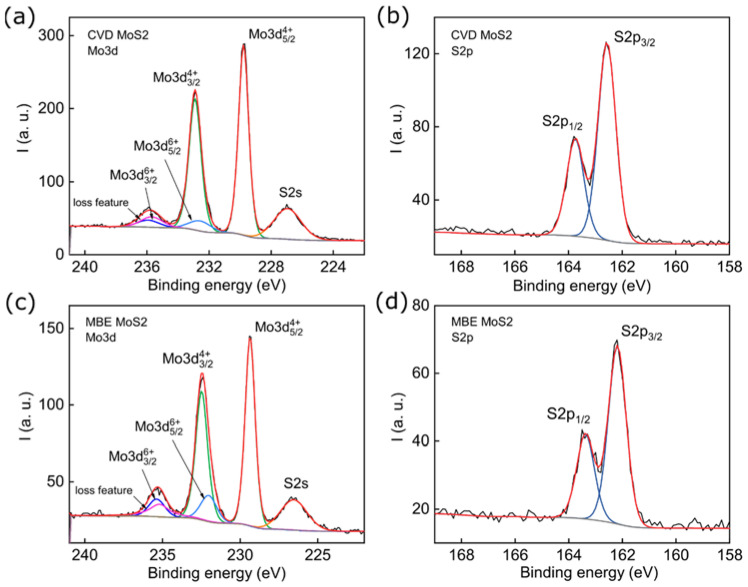
XPS characterization of CVD (**a**,**b**) and epitaxially (**c**,**d**) grown MoS_2_ monolayers on sapphire substrates. Decomposition of Mo3d (left) and S2p (right) core level signals into their constituents.

To assess the chemical purity of samples, we performed XPS measurements in Figure 3. A detailed study was performed in spectral ranges corresponding to bonds formed by Mo and S atoms. No impurities other than oxygen were found during XPS measurements.

The S2p spectra were described by a doublet with the S2p5/2 line position at 162.6 eV and a spin-orbit splitting of 1.2 eV for the CVD MoS_2_ sample and at 162.2 eV and the same spin-orbit splitting for the MBE MoS_2_ sample.

The Mo3d spectrum was decomposed of two doublets, with the more intense one corresponding to the Mo^4+^ state in the MoS_2_ compound. The less intense doublet corresponded to the Mo^6+^ state in the MoO_3_ compound. In addition, the S2s and “loss feature” lines were present in the spectra. The position of the Mo3d5/2 line for the CVD MoS_2_ and MBE MoS_2_ samples was 229.8 eV and 229.4 eV, respectively. The difference in the position of the lines could be caused by different levels of doping. The molybdenum fraction in the Mo6+ state for CVD MoS_2_ and MBE MoS_2_ samples was 0.10 and 0.18, respectively. The total atomic concentration ratio [S]/[Mo] for CVD MoS_2_ and MBE MoS_2_ samples was 1.8 and 1.7, respectively. No noticeable concentration of other impurities was observed in XPS measurements. The natural oxidation of single-layered MoS_2_ under ambient conditions has been previously reported [49] and the S vacancies are formed through oxidation spontaneously followed by an O substitution process since the oxidation is thermodynamically more favorable [50,51]. The results of XPS measurements indicate that the increased photoluminescence yield of MBE MoS_2_ might be due to the passivation of sulfur vacancies and crystallite boundaries by oxygen [52].

### 2.2. Dielectric Response Analysis

**Figure 4:**
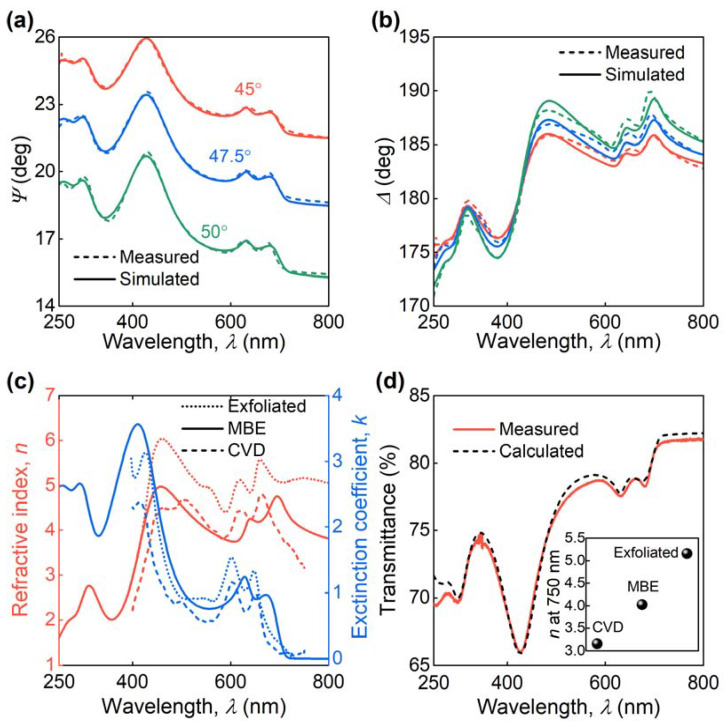
Optical properties of MBE MoS_2_. Plots of the measured and calculated MBE MoS_2_ ellipsometric parameters (**a**) *Ψ* and (**b**) Δ. (**c**) Optical constants (*n* and *k*) of epitaxial monolayer MoS_2_ grown on a sapphire substrate from SE analysis of panels (**a**,**b**). For the tabular data, see Table A1. For comparison, we added optical constants of CVD and exfoliated MoS_2_ from ref. [37,38], respectively. (**d**) Measured (red line) and calculated (black line) transmittance spectra of MBE MoS_2_ on sapphire matching perfectly within spectrophotometer accuracy (1%) except in the 250–270 nm range, where inaccuracy approaches 2% attributed to the low signal sensitivity of our ellipsometer in that interval. The inset is a refractive index of exfoliated, MBE, and CVD MoS_2_ at 750 nm.

The dielectric response of MBE MoS_2_ was determined using spectroscopic ellipsometry (SE), and the resulting spectra are shown in Figure 4a,b. The ellipsometric parameter Ψ clearly reveals MoS_2_ excitonic features, which we described through the Tauc-Lorentz oscillator model [38] with their resulting parameters collected in Table 1. Figure 4c depicts the corresponding dielectric function. Notably, the optical bandgap of the MBE sample equals 1.718 eV, while for CVD MoS_2_ this value is 1.744 eV [39]. This red-shift (0.026 eV) of optical bandgap is in agreement with the PL result (0.025 eV) from Figure 2a. We also recorded transmission spectra and compared them to the transfer matrix calculations [53] to verify the extracted dielectric response in Figure 4c and confirm its predictive capability. The measured and calculated transmittance spectra plotted in Figure 4d match perfectly, thus validating our result in Figure 4c.

Interestingly, the optical constants of MBE MoS_2_ are intermediate between CVD and exfoliated MoS_2_, as illustrated in Figure 4c. For example, at λ = 750 nm the refractive index of CVD, MBE, and exfoliated MoS_2_ are 3.2, 4.0, and 5.2, respectively. Therefore, the MBE growth technique allows getting closer to superior properties of exfoliated MoS_2_, but at a large scale. It makes MBE MoS_2_ a promising platform for scientific and industrial photonic applications.

**Figure 5:**
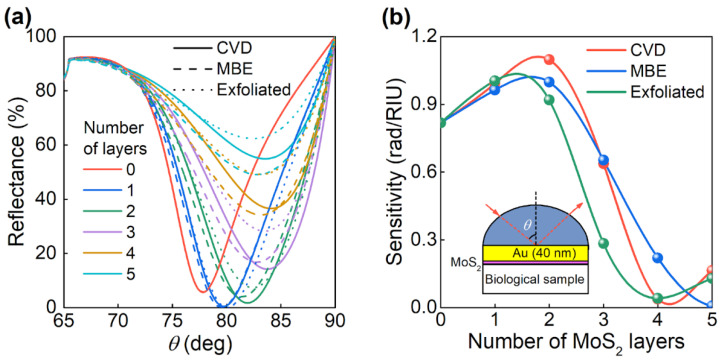
Surface plasmonic resonance (SPR) sensor based on SiO_2_/Au (40 nm) chip with CVD, MBE and exfoliated MoS_2_. (**a**) The reflectance of SPR sensor for different layer numbers of CVD, MBE, and exfoliated MoS_2_. (**b**) The dependence of the SPR sensor sensitivity on the MoS_2_ number of layers. The inset is a scheme of an SPR sensor.

The difference in the optical properties of MBE-synthesized MoS_2_ films from films fabricated by other methods must be taken into account when developing optical devices, since the difference in optical constants entails differences in characteristics of the device. To illustrate this, we consider a biosensor based on surface plasmon resonance in the Kretschmann scheme [54], in which a thin gold film covers a silicon oxide prism, and the change in the refraction index is detected by measuring the change of the resonant angle, at which the reflection from the scheme is minimal. To increase the sensitivity and coupling with the studied molecules, several layers of van der Waals material, such as graphene, are often deposited on the gold surface [55,56]. The addition of layers of molybdenum disulfide also improves the sensitivity of the biosensor. Figure 5 shows the dependence of the reflection coefficient on the angle of incidence (Figure 5a) and the dependence of the biosensor sensitivity on the number of MoS_2_ layers (Figure 5b). The calculations were performed for a wavelength of 635 nm, which is often used in optical biosensors, with a gold thickness of 40 nm. It turns out that even for a couple of MoS_2_ layers, both the dependence of the reflection coefficient on the angle of incidence and the sensitivity of the biosensor differ by more than 10% for MoS_2_ films obtained by different methods. As a result, fabrication technology provides an efficient way to control the dielectric function of MoS_2_, and hence, a method to tailor the optical response in photonic applications.

## 3. Materials and Methods

### 3.1. Materials

Isolated triangles with some full coverage areas of epitaxial MoS_2_ monolayer samples were purchased from 2d Semiconductors, Inc. (https://www.2dsemiconductors.com/ (accessed on 25 May 2021), Scottsdale, AZ, USA). Samples were grown in an MBE chamber at a base pressure of 8 × 10^−9^ Torr on a double-side polished c-cut sapphire. An extremely slow deposition rate of 5–100 atoms per second provides a single-crystal quality film deposition with a high crystallinity and reduced defect density. CVD-grown full area coverage monolayer MoS_2_ samples purchased from the SixCarbon Technology (http://www.6carbon.com/ (accessed on 25 May 2021), Shenzhen, China) were synthesized with atmospheric pressure chemical vapor deposition also on double-side polished c-cut sapphire.

### 3.2. Raman and Photoluminescence Characterization

The experimental setup used for Raman measurements was a confocal scanning Raman microscope Horiba LabRAM HR Evolution (HORIBA Ltd., Kyoto, Japan). All measurements were carried out using linearly polarized excitation at wavelengths 532 and 632.8 nm, 1800 lines/mm diffraction grating, and ×100 objective with a numerical aperture of 0.9. Meanwhile, we used unpolarized detection to have a significant signal-to-noise ratio. The spot size was ~0.43 µm. The Raman spectra were recorded with 0.75 mW (wavelength 632.8 nm) and 1.5 mW (wavelength 532 nm) incident power and an integration time of 3 s at each point. To compare CVD and epitaxial MoS_2_ PL response, we normalized PL spectra to A_1g_(Γ) Raman peak since they recorded simultaneously. The statistics were collected with at least 15 points for each sample, and the observed variation of the intensity for the spectra was less than 5%.

### 3.3. X-ray Photoemission Spectroscopy Characterization

For the detailed study of the CVD and epitaxial MoS_2_ monolayers grown on sapphire substrates, we performed measurements of the Mo3d and S2p5/2 level X-ray photoemission spectroscopy (XPS) spectra to reveal the difference in the elemental composition of two samples (Thermo Scientific K-Alpha, Waltham, MA, USA). Since the MoS_2_ samples on sapphire substrates were charged during the XPS measurements, the charge-compensation mode was used. The calibration was performed using the C1s line position at 284.5 eV.

### 3.4. Atomic Force Microscopy, Optical Visualization, Scanning Electron Microscopy

The roughness and homogeneity of the epitaxial MoS_2_ monolayer were measured by an atomic force microscope (NT-MDT Ntegra, Moscow, Russia). The MoS_2_ MBE fabricated sample was characterized immediately after unsealing without any pollution at ambient conditions, and thus these defects demonstrated in AFM and SEM in Figure 1d–f are the result of an MBE fabrication procedure. These defects might be Mo nucleation sites. The surface images (2400 × 2400 pixels) of the MoS_2_ samples were captured by an optical microscope (Nikon LV150, Tokyo, Japan) with a digital camera DS-Fi3. To investigate the MoS_2_ surface morphology, we additionally used the scanning electron microscope using the acceleration voltage of 3 kV (JEOL JSM-7001F, Tokyo, Japan).

### 3.5. Ellipsometry Characterization

Spectroscopic ellipsometry was conducted at several incident angles (45°, 47.5°, 50°) over a wide spectral range of 250 to 1700 nm (0.73–4.96 eV). An imaging ellipsometer in the rotation compensator mode was used for the measurements (Accurion nanofilm_ep4, Goettingen, Germany). We used the Tauc-Lorentz oscillator oscillator model for ellipsometry spectra analysis following the analysis algorithm developed by Ermolaev and colleagues [39,40]. The Tauc-Lorentz oscillator model is defined as:

$$\varepsilon_{2}=\{\begin{array}{c} \frac{1}{E}\cdot \frac{AE_{0}C{\left(E-E_{g}\right)}^{2}}{{\left(E^{2}-E_{0}^{2}\right)}^{2}+C^{2}E^{2}}\;\mathrm{for}\;E>E_{g}\\ 0\;\mathrm{for}\;E<E_{g} \end{array},$$

where *E* is the photon energy, *A* is the strength of the oscillator, *C* is the broadening term, *E*_g_ is the optical band gap, and *E*_0_ is the peak central energy. The real part ε_1_ of the dielectric function is derived from the expression of imaginary part ε_2_ of the dielectric function using the Kramers–Kronig integration.

## 4. Conclusions

To summarize, we have performed a detailed study of the structural and optical properties of monolayer MoS_2_ synthesized by MBE. We verified that the sample indeed contains a single-atomic layer of MoS_2_ through Raman and AFM measurements, while XPS measurements confirmed its high purity. The high crystallinity of the sample with a characteristic crystallite size of 6 μm was revealed by SEM, AFM imaging and dark-field microscopy. The quantum yield of MBE MoS_2_ by almost two times exceeds that of CVD MoS_2_, as demonstrated by PL measurements, which proves its superior quallities for active photonic applications. Finally, we have conducted accurate measurements of optical constants of MBE MoS_2_ by spectroscopic ellipsometry. The accuracy of the optical properties has been further verified by the measurement of the optical transmittance spectrum, which fully agrees with calculations based on the optical constants acquired by ellipsometry. Additionally, we demonstrated the significance of our accurate measurements for practical applications by comparing characteristics of SPR sensors calculated using the different constants. Even devices with only a few atomic layers of MoS_2_ demonstrate substantial differences in sensitivity and anticipated signal intensity when different sources of optical properties are used. Our results create a firm ground for photonic applications of atomically thin layers of transition metal dichalcogenides.

## Figures and Tables

**Table 1:** Tauc-Lorentz parameters of the oscillators (excitons) with $\varepsilon_{\infty }=0.54$ used to describe dielectric function of MBE MoS_2_.

Oscillator	*A* (eV)	*C* (meV)	*E*_g_ (eV)	*E*_0_ (eV)
#1	410	120	1.718	1.787
#2	84	88	1.832	1.955
#3	77	600	1.565	2.872
#4	80	731	2.856	4.070
#5	281	826	4.135	4.504

## Data Availability

The data presented in this study are available on request from the corresponding author.

## Appendix A

**Table A1:** Tabulated optical constants for epitaxial MoS_2_ monolayer on sapphire substrates.

*λ* (nm)	*n*	*k*
250	1.60684	2.61054
275	2.02921	2.55685
300	2.56575	2.58710
325	2.55812	1.89351
350	2.04339	2.16150
375	2.17595	2.89179
400	2.95143	3.48336
425	4.18875	3.38186
450	4.93334	2.40006
475	4.85958	1.51647
500	4.55210	1.04876
525	4.26415	0.83499
550	4.03556	0.75964
575	3.86255	0.77777
600	3.74944	0.90252
625	3.90919	1.22200
650	4.20291	0.90721
675	4.38497	0.96487
700	4.71574	0.32016
725	4.23306	0.00662
750	4.02327	0.00250
775	3.89866	0.00000
800	3.80981	0.00000
825	3.74192	0.00000
850	3.68740	0.00000
875	3.64225	0.00000
900	3.60403	0.00000
925	3.57116	0.00000
950	3.54254	0.00000
975	3.52215	0.00000
1000	3.49502	0.00000
1025	3.47888	0.00000
1050	3.45713	0.00000
1075	3.44403	0.00000
1100	3.42620	0.00000
1125	3.41536	0.00000
1150	3.40050	0.00000
1175	3.39140	0.00000
1200	3.37883	0.00000
1225	3.37110	0.00000
1250	3.36035	0.00000
1275	3.35371	0.00000
1300	3.34443	0.00000
1325	3.33867	0.00000
1350	3.33059	0.00000
1375	3.32556	0.00000
1400	3.31847	0.00000
1425	3.31404	0.00000
1450	3.30779	0.00000
1475	3.30387	0.00000
1500	3.29832	0.00000
1525	3.29482	0.00000
1550	3.28987	0.00000
1575	3.28674	0.00000
1600	3.28674	0.00000
1625	3.27949	0.00000
1650	3.27549	0.00000
1675	3.27296	0.00000
1700	3.26934	0.00000
